# Enhancing quality of life measurement: adapting the ASCOT easy read for older adults accessing social care

**DOI:** 10.1007/s11136-024-03791-0

**Published:** 2024-09-26

**Authors:** James Caiels, Stacey Rand, Rasa Mikelyte, Lucy Webster, Elizabeth Field, Ann-Marie Towers

**Affiliations:** 1 Personal Social Services Research Unit, University of Kent, Canterbury, UK; 2 Centre for Health Services Studies, University of Kent, Canterbury, UK; 3 Kent and Medway NHS and Social Care Partnership Trust, Gillingham, UK

**Keywords:** Quality of life, Easy read, Dementia, Older adults, Social care, Co-production

## Abstract

**Purpose:**

This study aimed to adapt and assess the content validity of the ASCOT Easy Read (ASCOT-ER) for older people accessing social care.

**Methods:**

A co-production working group of 8 older social care users and their supporters was established to evaluate the comprehensibility and relevance of the ASCOT-ER images, wording and layout. Changes made by the working group were iteratively tested using cognitive interviewing techniques (think aloud) with 25 older social care users not able to self-complete the original ASCOT.

**Results:**

Co-research with people with dementia and their supporters was critical to the development of an effective and accessible tool. Issues identified with comprehension, recall, judgement and response were addressed through iterative adjustments to design, layout and wording. An unexpected finding was that illustrations were disliked or disregarded by the majority of people, and, in particular, those living with dementia. This result contrasts with the typical assumption of easy read approaches, where illustrations are expected to enhance comprehension.

**Conclusion:**

The ASCOT-ER measure for older people is suitable for older people using social care services with mild to moderate dementia, mild cognitive impairment and other age-related needs. The revisions applied were designed to improve comprehension, judgement and response for this group and even those who were most cognitively impaired experienced fewer issues by the final round of testing. Nonetheless, some prompting was still required, particularly for those with higher levels of cognitive impairment and it is likely that some respondents will require the questionnaire to be administered in an interview format.

**Supplementary Information:**

The online version contains supplementary material available at 10.1007/s11136-024-03791-0.

## Introduction

In 2022/23, around half a million older adults in England, aged 65 or over, received publicly-managed long-term care support and services (also known as *social care* in the UK) [1]. This trend is expected to increase due to projected rises in complex multi-morbidity over the next 15 years [2]. In England, the wider UK and internationally, there has been an interest in how to measure the impact of social care services on people’s lives to determine the quality and cost-effectiveness of support. The Adult Social Care Outcomes Toolkit (ASCOT) (www.pssru.ac.uk/ascot) was developed to measure the social care related quality of life (SCRQoL) of adults using social care services [3, 4]. It has demonstrated good psychometric properties across diverse populations [3–5]. It has been recommended for economic evaluation of long-term care for older adults [6] and applied in adult social care practice, research and evaluation, both in England [7–9] and internationally [10, 11].

Some people who use social care services find conventional methods of data collection inaccessible for a variety of reasons [12]. However, some older people living in the community with conditions such as mild-to-moderate dementia, cognitive impairment and other age-related needs, who have capacity to consent, but are not able to respond to standard format questionnaires, may be able to self-report quality of life (QoL) with help [13] or adapted formats with simplified language or images [14].

Alongside other adapted versions designed to support inclusion of diverse adults in social care data collections, an adapted version of ASCOT using ‘easy read’ principles (ASCOT-ER) was co-produced with people with intellectual and developmental disabilities (IDD) or autism, and was found to be feasible, valid and internally consistent in survey data collection [15, 16]. This version consists of nine items, which cover eight QoL domains that can be supported by social care support: Personal cleanliness and comfort, Accommodation comfort and cleanliness, Food and drink, Safety (inside and outside of the home), Social participation, Occupation (‘doing things I value and enjoy’), Control over daily life, and Dignity. Each item is scored on a four-level scale, from ‘ideal state’ to ‘high-level needs’. An overall score can be generated from the sum of item scores from 0 (lowest QoL) to 24 (highest QoL) or by applying preference weights from − 0.17 to 1 (worst to best QoL), which can be used to evaluate and compare the quality and effectiveness of social care services.

The ASCOT-ER was used in an Australian qualitative study to determine its acceptability and feasibility for community-dwelling older adults with cognitive impairment [17]. This study found that ASCOT-ER was comprehensible and meaningful, with some adaptation, but also noted that the applied methodology of cognitive interviewing, which is a structured interview that asks respondents to ‘think aloud’ with probes to focus on aspects of survey questions [18], appeared to support people in answering the questions [17]. Despite the preliminary evidence from this study, consultations with older people in England indicated that the measure was not suitable for older adults. This particularly applied to the ASCOT-ER images, which had been previously co-designed and tested in focus groups or interviews with adults with IDD and/or autism, the majority of whom were aged 18–59 years [16]. Further work was required to co-produce an adapted version of the ASCOT-ER with older adults and assess its relatability, suitability, acceptability and meaningfulness.

Therefore, the aim of this study was to adapt and test a revised ASCOT-ER through a study based on co-design and cognitive interviews with older adults in England. Specifically, the study objectives were, first, to adapt the ASCOT-ER with simplified wording, layout and images around the criteria of making it *comprehensible*, *comprehensive* and *relevant* for older people using social care support and services. This included those with mild-to-moderate dementia, mild cognitive impairment, and other age-related needs that may make self-report with a standard questionnaire unfeasible. Second, we sought to establish whether the adapted version was *comprehensible*, *comprehensive* and *relevant* and, where there were identified issues with *comprehension, recall, judgement* and *response* [18, 19], to establish whether these could be mitigated by design, layout or wording of the questionnaire.

## Methods

The study had two phases which overlapped: (1) co-design of an adapted ASCOT-ER with a working group of older adults and (2) evaluating the *comprehension, recall, judgement* and *response* to the adapted ASCOT-ER through three rounds of cognitive interviews. In-between each round, the evidence gathered from the interviews were reviewed by the research team and working group, to agree on any changes to be tested in the subsequent round.

### Co-design with the working group

The working group (WG) comprised eight older people, predominantly living with dementia, and their supporters/carers. Members of the working group were recruited from a variety of local dementia, engagement and empowerment project (DEEP) groups, based at Kent and Medway NHS and Social Care Partnership Trust (KMPT), who were collaborators in the study. A KMPT clinical psychologist (and co-author) facilitated this recruitment. In total, eight WG meetings were held between 30th May 2022 and 5th Feb 2024. All meetings were well attended, with individuals occasionally absent due to other commitments or illness. Meetings were attended by at least two, and up to four members of the research team.

The WG played an integral and key role in the iterative adaptation and development of the revised ASCOT-ER measure.

In the initial meetings (1–3), members were invited to comment on the ASCOT-ER previously developed for people with IDD and/or autism [17] and co-design an adapted version to be used in the cognitive interviews. Three further meetings (4–6) were held after each round of cognitive testing. Members of the research team reported summary findings back to the group and presented possible changes to consider that may help overcome any identified issues related to comprehension, judgement or response. All members were invited to consider these proposals and also make additional suggestions. All potential changes were considered and discussed by the group until consensus was reached. These changes were then implemented and taken forward into the next round of cognitive interviews. All members made a substantial contribution in each meeting, which was chaired by a member of the research team experienced with working with people living with dementia (PLWD).

WG meetings 6–8 were used to develop and produce a lay summary of the study, accessible for PLWD.

### Cognitive interviews

#### Recruitment of participants

Participants for the cognitive interviews were recruited across Kent, Medway, Surrey, Sussex and Greater London, through a combination of approaches. These included via Join Dementia Research (JDR), which is an opt-in national research volunteer panel (www.joindementiaresearch.nihr.ac.uk), the DETERMIND study (a study of dementia care quality, www.determind.org.uk), in which people had opted in to be contacted about other related research projects, and via a homecare provider organisation.

Inclusion and exclusion criteria were applied. Inclusion criteria were: individuals aged 65 years or over, using community-based social care services (e.g. homecare, day centre, personal budget, information and advice, or support group), living in their own or another person’s home. Exclusion criteria were: not using any social care support or service, advanced dementia, lack capacity to consent to the cognitive interview, and care home residents. We also used an additional short ‘eligibility’ check to screen for people who would struggle to self-complete the standard ASCOT self-completion (SCT4) questionnaire, and also to check potential participants were in receipt of social care or support (online resource 1).

Respondents were asked a brief set of demographic questions by the researcher before commencing the cognitive interview (online resource 2). They were also asked to complete the Mini-Cog survey (www.mini-cog.com), a short tool to assess signs of cognitive impairment.

#### Participant consent and ethical approval

Potential participants were sent or given a letter of invitation and information sheet and asked to register interest in the study via email or telephone call with the research team. After agreeing to participate, the interview was scheduled at a mutually convenient time and location. This was usually the person’s home, face-to-face, but was occasionally at a local organisation’s centre. At the interview, participants were asked again if they were happy to take part and asked to sign a consent form. Interviews lasted 60–90 min, including the administration of the demographic questionnaire and Mini-Cog test. The cognitive interview was audio-recorded for transcription.

Ethical approval to conduct the study was granted by the Coventry and Warwick Research Ethics Committee on 14th December 2022 (Reference: 22/WM/0234).

#### Procedure and review

Three rounds of cognitive interviews were conducted by three researchers (JC, SR, LW), one male and two female interviewers, all of whom were trained in conducting interviews with older adults with mild cognitive impairment or dementia and their family carers. In some instances (4), a spouse or partner was present at the participant’s request. In these cases, interviewers remained vigilant to potential bias and influence on the interview process.

The interview was introduced to explain that the purpose was to help us improve the design of the questionnaire, not to ‘test’ the ability of the participant. Participants were asked to read and complete each question. While doing so, they were invited to ‘think aloud’ [20] to say what they were thinking. This method is effective for identifying response issues and has been successfully applied in similar contexts with outcome measures such as EQ-5D, EQ-HWB, ASCOT, and QOL-ACC [21]. The researcher asked follow-up questions (known as ‘probes’) to explore comprehension, how and why the person chose particular answers, how they indicated their answer, and to explore any potential issues with the questionnaire content, layout and design (see online resource 3 for the full interview schedule).

**Fig. 1:**
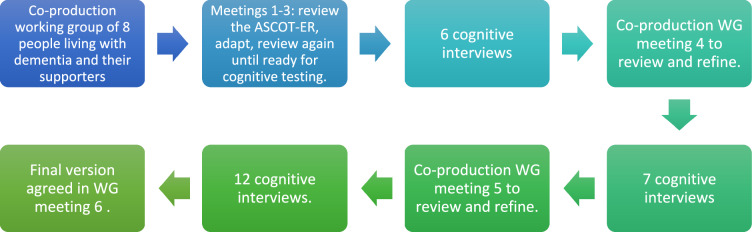
Summary of data collection and review process

Each round of interviews was with between 6 and 12 participants (see Fig. 1). This allowed iterative testing and adaptation of the questionnaire to improve its content and layout, with particular attention to any issues related to comprehension, judgement (weighing up response options) and indicating a response.

Interviews were audio recorded. The researchers also took field notes, which were combined after each round of cognitive interviews to identify significant or recurring issues. These were initially reviewed by the research team and any areas for revision agreed, e.g. where difficulties had been encountered by multiple participants.

#### Data analysis

Interview audio recordings were transcribed verbatim. Transcripts were coded in Nvivo software by three interviewers. The coding framework was based on Tourangeau’s four stage model of item response [22]: comprehension, (understanding of the domain/question descriptor); recall, (ability to recall appropriate/relevant information); judgement, (ability to assess information and form a response); and response mapping, (mapping a verbal answer to one response option). An additional code was included to record when participants required prompting from the interviewer, either because they became ‘stuck’ on a question, or needed reorienting or help understanding what was being asked [23].

While such formal coding of verbatim transcripts is not required for the analysis and application of cognitive interviews to improve survey design [18], the coding was applied to ensure no issues were missed from those identified in interviewers’ field notes. An ‘issue’ was taken to refer to the participant requiring prompting or difficulties with one of the four cognitive processes in Tourangeau’s four stage model [22]. The latter included non-response due to inability to decide on and/or select one response option (as required by the instructions) or evidence of misunderstanding the question (text or images) or response options, as intended to be understood by the developers. The coding was also used to quantify the number of issues encountered with questions (including type) during each round of testing. To ensure consistency and accuracy, the coding was discussed in regular meetings between the three interviewers during and between each round of interviews. Any concerns or inconsistencies were resolved within these meetings, or, if necessary, the wider research team were involved. Coding examples can be seen in online resource 4.

## Results

### Co-design with the working group

Throughout WG meetings 1–3, and prior to commencing the cognitive interviews, a number of revisions and subsequent refinements were made to the ASCOT-ER previously developed with adults with IDD and/or autism. First, members of the WG (meeting 1) unanimously shared the view that the illustrations were: not relevant or relatable (e.g. people depicted working); reinforced stereotypes (e.g. ‘older people need help’); and could alienate respondents. Additionally WG members disliked the general style of the illustrations describing them as “too busy, not big enough”, with too much detail making them difficult to decipher, especially for one vision impaired member. One aspect that WG members thought to be helpful were the ‘emoji style’ happy/sad faces that accompanied response options. In response to these observations, the illustrations were replaced with alternatives developed by colleagues engaged in parallel efforts to adapt the ASCOT-ER for the German/Austrian older population [24]. These were then refined by the research team in collaboration with the WG.

The working group also made changes to the wording of questions and response options. It was agreed that longer response options could be condensed, as whilst these fuller descriptions were helpful for people with IDD and/or autism, they were not necessary or helpful for older people. Qualifiers provided alongside response options (such as ‘it is ok’ or ‘it is bad’) were also removed. Other words were changed or added to support comprehension and relevance (e.g. changing having ‘choice’ to ‘control’; changing ‘being presentable’ to ‘being clean and comfortable’). Some wording or their order of presentation was amended to reflect the concerns of older people (e.g. moving ‘falling or getting hurt’ to the first bullet point for the Safety question), see online resource 5 for an example question. Interestingly, some changes meant that the wording of questions, as developed for the ASCOT-ER, returned to a version closer to the standard self-completion version (ASCOT-SCT4), which was originally developed and tested with older people using homecare services who could self-report [5, 25]. Online resource 6 shows the provisional questionnaire to be used in the Round one cognitive interviews.

### Cognitive interviews—sample

Table 1 shows the demographic characteristics of our sample, which consisted of 25 respondents in total. Of these, 52% were female and 80% were white British, with 20% from a Black or minority ethnic group. Most respondents (88%) were living in their own home with at least one other person (68%). Respondents were accessing multiple and varied types of care. A majority (56%) were using or engaging in some kind of community activity or support. This included, e.g., attending a day care or community centre for educational workshops, fitness classes, other cultural or hobby activities, or support groups. Almost half (44%) were using homecare and 40% were using some kind of equipment (e.g. wheelchair) and/or home adaptation (e.g. grab rail). Most respondents paid for their own care and support in full (56%), while others did not pay for their care (i.e. publicly funded or voluntary sector) (36%) or paid only a contribution (8%).

**Table 1:** Sample characteristics

Age (Mean, Std. Dev, Range)	Overall, n = 25 (%)	Round 1, n = 6 (%)	Round 2, n = 7 (%)	Round 3, n = 12 (%)
	(78, (SD 7.31), 65–91)	(82, (SD 6.42), 72–89)	(80, (SD 4.91), 75–88)	(75, (SD 7.58), 65–91)
*Gender*				
Male	12 (48%)	2 (33%)	3 (43%)	7 (58%)
Female	13 (52%)	4 (67%)	4 (57%)	5 (42%)
*Ethnicity*				
White British	20 (80%)	6 (100%)	6 (86%)	8 (67)
White other	1 (4%)		1 (14%)	
Black, Black British, Caribbean or African	2 (8%)			2 (17%)
Asian or Asian British	1 (4%)			1 (8%)
Specified Other	1 (4%)			1 (8%)
*Housing*				
Own home	22 (88%)	5 (83%)	6 (86%)	11 (92%)
Rented home	2 (8%)	1 (17%)		1 (8%)
Assisted living	1 (4%)		1 (12%)	
*Living arrangement*				
Living with at least one other	17 (68%)	5 (83%)	4 (57%)	8 (67%)
Living alone	8 (32%)	1 (17%)	3 (43%)	4 (33%)
*Social care services*				
Homecare	11 (44%)	5 (83%)	3 (43%)	3 (25%)
Live-in care	3 (12%)		1 (12%)	2 (17%)
Day centre	2 (8%)			2 (17%)
Community activities	14 (56%)	2 (33%)	2 (29%)	10 (83%)
Equipment	10 (40%)	3 (50%)	4 (57%)	3 (25%)
Home adaptations	10 (40%)	3 (50%)	4 (57%)	3 (25%)
Other^a^	6 (24%)	2 (33%)	3 (43%)	1 (8%)
*Pay for own care?*				
No	9 (36%)		3 (43%)	6 (50%)
Yes, a contribution	2 (8%)	1 (17%)		1 (8%)
Yes, in full	14 (56%)	5 (83%)	4 (57%)	5 (42%)

^a^Includes: Monthly support group for carers/PLWD; Age UK support/Advice; Singing for Dementia; Lifeline; Alzheimer’s support group

### Cognitive interviews

The changes made to the ASCOT-ER after each round of interviews are summarized in Table 2.

**Table 2:** Modifications after each round of cognitive interviews

Attribute/item	Rationale for modification
*After Round one*	
Food and drink	N/A
Accommodation	N/A
Personal cleanliness and comfort	Illustration amended. Water from the shower removed, and other water made lighter to improve clarity
Safety inside	N/A
Safety outside	N/A
Control	N/A
Social Participation	Question relocated from question 7 to question 5 in the questionnaire to avoid conflation of the Social participation and Occupation questions
Occupation	N/A
Dignity	N/A
All items	Tick boxes relocated to the left hand side of all response options. This is to improve read-across ‘Tick one box’ has been amended to ‘tick only one box’. Example given amended to use an actual box (rather than a tick in parenthesis). This is to help responders follow the instruction and show what they need to do
*After Round two*	
Food and drink	N/A
Accommodation	N/A
Personal cleanliness and comfort	N/A
Safety inside	N/A
Safety outside	Question stem amended from: *‘This question is about feeling safe when you go out in your local area’* to: *‘This question is about feeling safe when you go out. Think about the places you usually go, and who you go with’* to improve respondents’ comprehension and understanding of what to consider
Control	Question stem amended from: *‘Think about all the things you do during the day. This could be your free time, volunteering or helping others, and doing housework’* to *‘Think about all the things you do during the day. This could be helping others, doing housework, or leisure activities like hobbies, watching TV and reading’* to improve respondents’ comprehension and understanding of what to consider
Social Participation	N/A
Occupation	N/A
Dignity	Question amended from: *‘How do you feel about the way your paid support treat you?’* to: *‘How do you feel about the way your paid support treat you? By paid support we mean anyone that is paid by you, or anyone else, including the council, to support you’* to improve comprehension and clarify the meaning of ‘paid support’
All items	Two versions used (offered) in round three testing: one without illustrations, one with illustrations (excluding response option ‘faces’)
*After Round three*	
Food and drink	Bullet points removed from question stem to prevent respondents getting ‘stuck’ at these points and misunderstanding that each bullet point needed to be responded to or answered as a question
Accommodation	N/A
Personal care	N/A
Safety inside	Bullet points removed from question stem to prevent respondents getting ‘stuck’ at these points and misunderstanding that each bullet point needed to be responded to or answered as a question
Safety outside	Bullet points removed from question stem to prevent respondents getting ‘stuck’ at these points and misunderstanding that each bullet point needed to be responded to or answered as a question
Control	N/A
Social Participation	N/A
Occupation	Bullet points removed from question stem to prevent respondents getting ‘stuck’ at these points and misunderstanding that each bullet point needed to be responded to or answered as a question
Dignity	Question amended from: *‘How do you feel about the way your paid support treat you? By paid support we mean anyone that is paid by you, or anyone else, including the council, to support you’* to: *‘How do you feel about the way your paid support treat you? By paid support, we mean any person, groups, activities or service that is paid to support you. This includes homecare, befrienders or visitors, social activities or support groups, or help from organisations, like Age UK.’* This is to further clarify the meaning of ‘paid support’ and improve comprehension and understanding of what to consider
All items	Underlined ‘only one box’ to help instruction All illustrations removed. After findings in round 3 of cognitive interviews. WG members (PLWD) agreed that these were unhelpful and provided distraction to the question text

#### Round one

The illustration for Personal cleanliness and comfort was revised to remove visual clutter by eliminating the depiction of water from the shower, and lightening the remaining water for clarity.

The Social participation question was relocated from question 7 to question 5 in the questionnaire. The aim was to prevent conflation of Social participation with the Occupation question. During round 1 this led some respondents to narrowly interpret Occupation (‘doing things I value and enjoy’) to consider only social activities, overlooking other activities, e.g. reading or watching TV. Changing the question order mitigated this issue in the subsequent rounds of cognitive interviews.

Further refinements included moving the tick boxes to the left-hand side and revising the instruction to emphasise ‘tick *only* one box’ (emphasis in *italic* added). The instructional example was also modified to precisely mirror the available response options. The previous indication of a tick within parenthesis—(✔)—was replaced with a tick within a box, to align with how respondents were to indicate their answer.

All changes made were agreed by WG members in meeting 4. Online resource 7 shows the changes made for Round two.

#### Round two

Following the second round of cognitive interviews, several further adjustments were made (see Table 2). The Safety outside question stem was revised to focus on respondents’ typical outings, rather than hypothetical scenarios, in which they would not usually find themselves e.g. out alone after dark. Similarly, the Dignity question stem was edited to define the term ‘paid support’.

Findings from round 2 revealed dissatisfaction with the illustrations. Several participants described these visuals, alongside happy/sad faces for the response options, as “*silly*”, “*unhelpful*”, or said the images were unclear and did not convey the intended meaning. The research team agreed to further probe this issue in remaining interviews. Most respondents indicated that they relied solely on the text to understand the question, with illustrations either being disregarded or perceived as a hindrance.

To address this, two versions, with and without illustrations, were introduced for testing in Round 3. Respondents were asked to choose their preferred format and offered the alternative version if they encountered difficulty during the interview.

All changes made for Round three were agreed by WG members in meeting 5. These are shown in online resource 8.

#### Round three

**Fig. 2:**
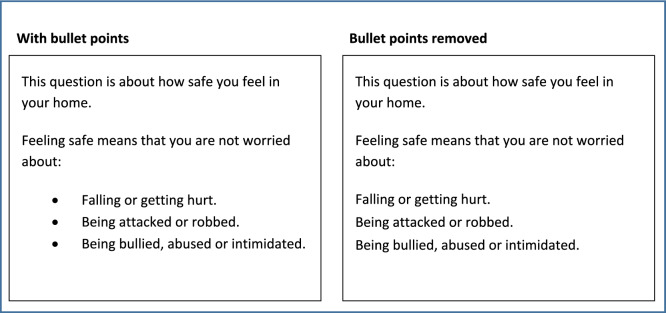
Example of bullet of points removed from question

The third and final round of cognitive interviews identified some remaining issues, which required further adjustment (see Table 2). Some respondents became ‘stuck’ at the bullet point lists in the following questions: Food and drink, Safety (inside/outside), and Occupation. They mistakenly thought they had to respond to each bullet point, as separate questions. Given time, many respondents eventually realised that they were not separate questions. However, the bullet point format seemed to hinder, rather than support, comprehension and response. The bullet points were removed to provide the list in a single sentence, see Fig. 2.

For some respondents, difficulties persisted regarding the concept of ‘paid support’ in the Dignity question. To enhance clarity, examples were added to guide respondents (Table 2).

Despite earlier adjustments, a small number of respondents continued to struggle with selecting only one response option. To emphasise this requirement, the last three words of the instruction to ‘tick only one box’ was underlined, a change suggested and endorsed by the WG.

During round 3, most respondents (n = 8 of 12) opted for the non-illustrated version, when given the choice. Of the 4 respondents who chose the version with illustrations, one said the images were not useful when asked. The remaining three were judged by the interviewer (based on close observation) not to have directly referred to or used the illustrations to support comprehension. Working group members, in particular PLWD, concurred that the illustrations were at best superfluous, and at worst unhelpful and detracted from the question text.

Although it is unusual to make further changes in the final round of cognitive interviews, we were constrained by time and resources from undertaking further cognitive interviews. Instead, it was unanimously agreed with working group members (in WG meeting 6) to remove illustrations from the final version of the questionnaire. For this version see: www.pssru.ac.uk/ascot/ascot-er-op/.

### Response issues by type and cognitive impairment

Table 3 shows the frequency of response issues by cognitive impairment (as measured by the Mini-Cog score) and response stage based on Tourangeau’s model (comprehension, recall, judgement and response) [22] and also whether the person needed prompting to complete the question.

**Table 3:** Frequency of issues by cognitive impairment and domain question (round one)

	Round one (all) (n = 6)					Round one (MC 0–2) (n = 4)					Round one (MC 3–5) (n = 1)				
	Com	Rec	Jud	Res	Pro	Com	Rec	Jud	Res	Pro	Com	Rec	Jud	Res	Pro
Food and drink		1	1	4	2				3	2					
Accommodation	1		1	1	2	1		1		1					
Personal care	1		1	2		1		1	1						
Safety inside	1			2	1	1			2	1					
Safety outside															
Control	1		2	4	2			1	2	1				1	
Social	1		1	3	2	1		1	3	2					
Occupation	1	1	2	4	1	1	1	2	3	1					
Dignity	2	1	1	2	1	1	1	1	2	1					
Total	8	3	9	22	11	6	2	7	16	9	0	0	0	1	0
Grand total	53					40					1				

*Com* Comprehension, *Rec* Recall, *Jud* Judgement, *Res* Response Mapping, *Pro* Prompt required

In round one, a total of 53 issues were identified, with the majority occurring during response, followed by the judgement stage. All participants experienced at least one issue. Of interest are the subsequent columns, which stratify these issues by Mini-Cog scores. The first group comprises individuals who scored 0–2 on the Mini-Cog test (MC 0–2), indicating a higher likelihood of cognitive impairment due to dementia (www.mini-cog.com). In contrast, column three represents individuals scoring 3–5, suggesting a lower likelihood of cognitive impairment due to dementia. The disparity between the two groups is noteworthy. However, it is important to note the small sample size, and that not all participants completed the Mini-Cog assessment due to fatigue, feeling unwell, time constraints, or personal choice.

Following the modifications after round one, round two interviews showed a reduction in the occurrence of issues. As before, the response mapping stage remained the most challenging. Notably, among people with poorer cognition, the number experiencing issues dropped from 40 in round one to 3 in round two. This is approximately 10 issues per person in round one, compared to 1.5 issues per person in round two. Again, we acknowledge the small sample size, but it is important that we see the number of issues reduce per round, as we would expect if we are making ‘successful’ changes to the questionnaire (Table 4).

**Table 4:** Frequency of issues by cognitive impairment and domain question (round two)

	Round two all (n = 7)					Round two MC 0–2 (n = 2)					Round two MC 3–5 (n = 4)				
	Com	Rec	Jud	Res	Pro	Com	Rec	Jud	Res	Pro	Com	Rec	Jud	Res	Pro
Food and drink				2	1				1					1	1
Accommodation															
Personal care															
Safety inside															
Safety outside	1			1							1			1	
Control	1			1		1			1						
Social															
Occupation				2	1									2	1
Dignity															
Total	1	0	0	5	2	1	0	0	2	0	0	0	0	3	2
Grand total	10					3					7				

*Com* Comprehension, *Rec* Recall, *Jud* Judgement, *Res* Response Mapping, *Pro* Prompt required

In round three we see this ‘trend’ continuing, descriptively, with the caveat of a limited sample size. Nonetheless, it offers insight into respondents’ experiences when engaging with the questionnaire. Notably there were 3 issues observed among 4 people in the MC 0–2 group, and 1 issue observed among 3 people in the MC 3–5 group. While the sample prohibits any meaningful statistical analysis, these findings provide descriptive insight into the third round interview findings, which had fewer issues identified. Response mapping emerges as a key issue across both groups, while individuals with Mini-Cog scores of 3–5 exhibited fewer issues and demonstrated less need for prompting. Consequently we anticipate that the majority of people, particularly those with higher cognitive function, will be able to complete the measure satisfactorily (Table 5).

**Table 5:** Frequency of issues by cognitive impairment and domain question (round three)

	Round three all (n = 12)					Round three MC 0–2 (n = 4)					Round three MC 3–5 (n = 3)				
	Com	Rec	Jud	Res	Pro	Com	Rec	Jud	Res	Pro	Com	Rec	Jud	Res	Pro
Food and drink					3										
Accommodation															
Personal care															
Safety inside															
Safety outside					1					1					
Control															
Social															
Occupation				1	1				1						
Dignity	2				1					1	1				
Total	2	0	0	1	6	0	0	0	1	2	1	0	0	0	0
Grand total	9					3					1				

*Com* Comprehension, *Rec* Recall, *Jud* Judgement, *Res* Response Mapping, *Pro* Prompt required

## Discussion

This study applied a co-productive and cognitive interview approach to adapt the existing ASCOT-ER designed and tested with people with IDD and/or autism, to modify its wording, layout, and images to enhance its comprehensibility for older people accessing social care services. The adaptation aimed to accommodate those with mild to moderate dementia, mild cognitive impairment, and other age-related needs, to support people to self-report using a questionnaire format. Issues identified with comprehension, recall, judgement and response were addressed through iterative adjustments to design, layout and wording.

An unexpected finding was that illustrations were disliked or disregarded by the majority of people, and, in particular, those living with dementia. This result contrasts with the typical assumption of easy read approaches, where illustrations are expected to enhance comprehension. While previous research developing the ASCOT-ER for individuals with IDD and/or autism supported this assumption, here our study revealed a different outcome. Some people regarded or found the illustrations to be an ‘active distraction’, prompting a compelling argument for their removal. Furthermore, insight from a recent study of the same/similar illustrations with older people, which utilised eye-tracking software, showed individuals only briefly scanning illustrations, suggesting minimal impact on comprehension [24].

Whilst the findings support the removal of images, it is interesting to note that layout and visual design were important in facilitating comprehension. This includes, for example, the left alignment of text and using lists without bullet points. This highlights the distinction between adding illustrations for comprehension, as observed in individuals with IDD and/or autism, and the layout of text content to enhance accessibility and clarity for older people. Moreover, this finding underscores the crucial importance of co-designing tools with the specific target demographic, rather than assuming ‘read across’ from other groups. Without the valuable input from the WG, the decision to retain the illustrations might have persisted, based on the prior assumption that ‘images will help’, as informed by the ER literature that is mostly based on people with IDD. Therefore, collaborative efforts with the target population are essential in ensuring the development of effective and accessible tools.

The revised tool is primarily intended for individuals with mild to moderate dementia, mild cognitive impairment, and other age-related needs. When assessing the questionnaire with individuals with milder levels of cognitive impairment, as indicated by higher Mini-Cog scores, people encountered fewer issues. This suggests promising feasibility of the revised measure, particularly for those with intact to mild cognitive impairment. Respondents were able to comprehend the questions, make a judgement on their own situation, and indicate their response. For respondents with higher levels of cognitive impairment, they could still complete the measure, albeit with some form of assistance or support, as indicated in our findings by the higher incidence of prompting from the interviewer. It is important to note that this version is not intended to replace the ASCOT-ER (developed for adults with IDD and/or autism), or the standard ASCOT. Rather, it is part of a suite of tools (www.pssru.ac.uk/ascot) designed to measure the social care-related QoL of adults with a range of care and support needs, who use social care services (e.g. homecare, residential care). The suite of tools include different versions, which seek to promote accessibility and inclusion. This revised version (ASCOT-ER (OP)) is an addition to the toolkit, for older adults, aged 65 or over, with mild cognitive impairment, mild-to-moderate dementia or other ageing-related needs (see online resource 9 for a comparison of the ASCOT-ER, ASCOT-ER (OP)).

This study contributes to the broader literature on the application of easy read approaches [26, 27]. By employing a co-design and cognitive interview approach, this study demonstrates the use of inclusive design principles in adapting and developing self-completion of questionnaires among diverse populations, highlighting important differences between adult populations (e.g. people with IDD and people living with dementia) [28, 29].

The iterative rounds of cognitive testing and development undertaken in this study emphasise the value of a systematic approach in addressing issues related to comprehension, recall, judgement and response among older people with mild to moderate dementia, mild cognitive impairment, and other age related needs. This iterative process ensures the refinement of the questionnaire’s wording and layout to improve its overall usability and accessibility. Moreover, testing the revised measure with a sample of older people across a spectrum of cognitive abilities supports the potential use of the adapted tool with diverse older people accessing social care services [30].

Despite the study’s contributions, a number of limitations warrant consideration. First, the majority of respondents were homeowners and self-funding their care. Ideally, a more diverse sample, including more individuals receiving social care funded by local authorities would have strengthened the study. Also, some changes (e.g. removing bulleted lists) were made at the final stage of the study and although subject to input from our working group (including people living with dementia), they have not been subject to testing through cognitive interviewing. It will be important to consider the impact of these final changes when the measure is piloted with a larger sample.

## Conclusion

Our study represents significant enhancements of the ASCOT-ER measure for older people accessing social care services with mild to moderate dementia, mild cognitive impairment and other age-related needs. The revisions applied were designed to improve comprehension, judgement and response for this group. Nonetheless, some prompting was still required, particularly for those with higher levels of cognitive impairment, and it is likely that some respondents will require the questionnaire to be administered in an interview format. It will be important to establish the feasibility and psychometric properties of the revised measure in future work.

## Supplementary Information

Below is the link to the electronic supplementary material.

Supplementary file1 (PDF 413 KB)

Supplementary file2 (PDF 604 KB) 

Supplementary file3 (PDF 254 KB)

Supplementary file4 (PDF 104 KB)

Supplementary file5 (PDF 718 KB)

Supplementary file6 (PDF 1492 KB)

Supplementary file7 (PDF 1507 KB)

Supplementary file8 (PDF 1453 KB)

Supplementary file9 (PDF 245 KB)

## Data Availability

The participants of this study did not give written consent for their data to be shared publically.
