# Supplementary material for: Enhancing quality of life measurement: adapting the ASCOT easy read for older adults accessing social care
Source: Qual Life Res. 2024 Sep 26;34(1):189–200. doi: 10.1007/s11136-024-03791-0 (PMC11802674; doi:10.1007/s11136-024-03791-0)
Supplement: Supplementary file 1 — Supplementary file1 (PDF 413 KB) [file 11136_2024_3791_MOESM1_ESM.pdf]

ASCOT-ER Eligibility check

When the researcher contacts or is contacted by a potential participant, the researcher will conduct an eligibility check with them over the telephone before they arrange the interview. In this they will check that the person receives social care and whether the need support to fill our paperwork/answer questionnaires, and thus needs an easy read format of the ASCOT.

"Thank you for your interest in this study. I just need to check that you eligible to take part by asking you a few questions:

(1) Do you currently receive social care? [Prompt if needed: "for example, do you have support from care workers with washing, dressing, meals or keeping your house clean? You might call know it as home care"]

IF YES, ASK Q2

IF NO, double check and then explain they are not eligible: So just to confirm, you do not currently receive any help or support at home? I'm very sorry but to take part if the research you do need to be using social care services and support. Thank you for your interest in the study and hopefully there will be other opportunities to take part in other work in the future."

IF CONFUSED (may indicate lack of capacity): That's okay, don't worry. We are really grateful you got in touch with us. However, on this occasion we won't be able to include you in the study. Hopefully there will be other opportunities to be involved in research in the future and thank you again for getting in touch.

(2) "Do you normally need some help to fill out paperwork/ answer a questionnaire? By help, we mean help to read or understand the question, to think about the answer, or to write the answer."

IF YES: arrange the interview

IF NO, check and then explain they are not eligible: So just to confirm, you are able to manage your paperwork at home or fill out forms and things without any help? I'm very sorry but we are looking for people who find that kind of thing difficult for this study, so on this occasion you are not eligible to take part. Thank you for your interest in the study and hopefully there will be other opportunities to take part in other work in the future."

To be eligible, the person must answer yes to both questions.
