# Supplementary material for: Enhancing quality of life measurement: adapting the ASCOT easy read for older adults accessing social care
Source: Qual Life Res. 2024 Sep 26;34(1):189–200. doi: 10.1007/s11136-024-03791-0 (PMC11802674; doi:10.1007/s11136-024-03791-0)
Supplement: Supplementary file 2 — Supplementary file2 (PDF 604 KB) [file 11136_2024_3791_MOESM2_ESM.pdf]

Participant number (researcher to add):______

Adapting the Easy Read Adult Social Care Outcomes Toolkit (ASCOT-ER) for older social care users

Participant Characteristics Questionnaire

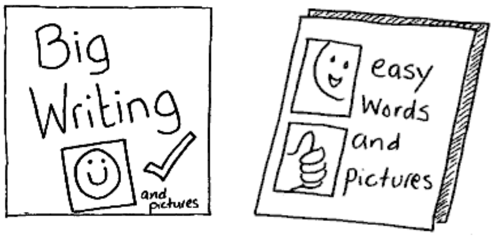

1. Which of the following describes how you think of yourself?

☐ Male

☐ Female

☐ In another way

☐ Prefer not to say

2. What is your age? ___________________

☐ Prefer not to say

ASCOT
adult social care outcomes toolkit

University of Kent

3. Which of the following describes your ethnicity (please tick one of the options)?

- [ ] White
- [ ] Mixed or multiple ethnic groups
- [ ] Asian or Asian British
- [ ] Black, Black British, Caribbean or African
- [ ] Other ethnic group - Please specify: ______________
- [ ] Prefer not to say

4. What kind of housing do you live in (please tick one of the options)?

- [ ] In a home I own
- [ ] In a home I rent / somebody else owns
- [ ] In assisted living / sheltered housing
- [ ] Prefer not to say

5. Do you live alone (please tick one of the options)?

☐ Yes, I live alone

☐ No, I live with other people

6. What kind of social care services do you use (please tick all of the options that apply)?

☐ Home care / domiciliary care

☐ Live-in care

☐ Day or drop-in centre

☐ Community activities

☐ Equipment (e.g. walking aids)

☐ Home adaptations (e.g. grab rail)

☐ Other - Please specify: ___________________

7. Do you pay for or towards care or support yourself?

- ☐ Yes, I pay for care and support with own money
- ☐ Yes, I pay a contribution towards care and support
- ☐ No, social services pay for all care and support
- ☐ No, I only use care and support that does not charge
- ☐ Other - Please specify: ________________
- ☐ Not sure / don’t know
