# Supplementary material for: Enhancing quality of life measurement: adapting the ASCOT easy read for older adults accessing social care
Source: Qual Life Res. 2024 Sep 26;34(1):189–200. doi: 10.1007/s11136-024-03791-0 (PMC11802674; doi:10.1007/s11136-024-03791-0)
Supplement: Supplementary file 3 — Supplementary file3 (PDF 254 KB) [file 11136_2024_3791_MOESM3_ESM.pdf]

Adapting the Easy Read Adult Social Care Outcomes Toolkit (ASCOT-ER) for older social care users

Cognitive Interview Schedule

Introduction to questionnaire

Thank you for agreeing to talk to us. My name is __________ and I work at the University of Kent in Canterbury.

This study aims to produce a new 'easy to read' version of a widely used questionnaire - the ASCOT (Adult Social Care Outcomes Toolkit). Some older people using social care find it difficult to complete the ASCOT because of memory problems or other problems they may have.

We have previously made an easy read version of the ASCOT for adults with learning disabilities. Now we want to make an easy read version for older people. This will include pictures and bigger writing.

I will ask you to look at the form and answer the questions. Think out loud as you go along. This means telling me what you think and why you have answered the way you did.

After each question, I will ask you:

- Why you chose a particular answer, and
- What you thought about the question, was it easy to understand the question, and what kinds of things were you thinking about when choosing your answer.

We are interested in how you understand the questions in the form and how easy or difficult it is to answer them. There are no wrong answers and this isn't a test.

Everything you tell us is "confidential" and will not be accessible to anyone other than the researchers on the project. However, if you do indicate during the questionnaire that you are being harmed, or anyone's safety or health is at risk, we will have to contact someone. This is the only time your answers could be linked to you.

I would like to record the interview. Only researchers working on this project will have access to the recording and any transcripts. All data will be securely stored. Are you happy for me to record the interview?

Do you have any questions?

Are you ready to start? If you need a break or you would like to stop, please let me know.

[start recording, if consented]

ASCOT domain: control over daily life

Let the participant read (out) the question and answers, and give an answer...

**Insert latest round version of the personal cleanliness domain question and answers to be used in each round of interviews**

Questions/probes (if needed):

- How would you answer this question? Which answer would you choose?
- Why? / What were you thinking about when you chose that answer? / Why did you choose this answer rather than …?
- Was it easy or difficult to answer the question? / Were there any words or phrases that were unclear?
- Could this question (the wording, picture or its layout) be made clearer? If so, in what way?
- What were you thinking about when you answered the question?

- [Note additional questions/probes as relevant for each round of interviews]

More Spontaneous Probes:

- I noticed you were spending some time with that question – can you tell me what you were thinking about?
- You answered that very quickly, why is that?
- You answered ______ - in your own words, what does that mean?
- How sure are you of your answer?
- How did you feel about answering this question?
- I noticed you were looking here (and there), what were you thinking?

ASCOT domain: personal cleanliness

Let the participant read (out) the question and answers, and give an answer.

**Insert latest round version of the personal cleanliness domain question and answers to be used in each round of interviews**

Questions/probes (if needed):

- How would you answer this question? / Which answer would you choose?
- Why? / What were you thinking about when you chose that answer? / Why did you choose this answer rather than …?
- Could this question (the wording, picture or its layout) be made clearer? If so, in what way?
- Was it easy or difficult to answer the question?
- What were you thinking about when you answered the question?

- [Note additional questions/probes as relevant for each round of interviews]

More Spontaneous Probes:

- I noticed you were spending some time with that question – can you tell me what you were thinking about?
- You answered that very quickly, why is that?
- You answered ______ - in your own words, what does that mean?
- How sure are you of your answer?
- How did you feel about answering this question?
- I noticed you were looking here (and there), what were you thinking?

ASCOT domain: food and drink

Let the participant read (out) the question and answers, and give an answer.

**Insert latest round version of the food and drink domain question and answers to be used in each round of interviews**

Questions/probes (if needed):

- How would you answer this question? / Which answer would you choose?
- Why? / What were you thinking about when you chose that answer? / Why did you choose this answer rather than …?
- Could this question (the wording, picture or its layout) be made clearer? If so, in what way?
- Was it easy or difficult to answer the question?
- What were you thinking about when you answered the question?

- [Note additional questions/probes as relevant for each round of interviews]

More Spontaneous Probes:

- I noticed you were spending some time with that question – can you tell me what you were thinking about?
- You answered that very quickly, why is that?
- You answered ______ - in your own words, what does that mean?
- How sure are you of your answer?
- How did you feel about answering this question?
- I noticed you were looking here (and there), what were you thinking?

ASCOT
adult social care outcomes toolkit

University of Kent

ASCOT domain: accommodation

Let the participant read (out) the question and answers, and give an answer.

**Insert latest round version of the accommodation domain question and answers to be used in each round of interviews**

Questions/probes (if needed):

- How would you answer this question? / Which answer would you choose? How do you think the person would answer?
- Why? / What were you thinking about when you chose that answer? / Why did you choose this answer rather than …?
- Could this question (the wording, picture or its layout) be made clearer? If so, in what way?
- Was it easy or difficult to answer the question?
- What were you thinking about when you answered the question?

- [Note additional questions/probes as relevant for each round of interviews]

More Spontaneous Probes:

- I noticed you were spending some time with that question – can you tell me what you were thinking about?
- You answered that very quickly, why is that?
- You answered ______ - in your own words, what does that mean?
- How sure are you of your answer?
- How did you feel about answering this question?
- I noticed you were looking here (and there), what were you thinking?

ASCOT
adult social care outcomes toolkit

University of Kent

ASCOT domain: safety in the home

Let the participant read (out) the question and answers, and give an answer.

**Insert latest round version of the safety in the home domain question and answers to be used in each round of interviews**

Questions/probes (if needed):

- How would you answer this question? / Which answer would you choose?
- Why? / What were you thinking about when you chose that answer? / Why did you choose this answer rather than …?
- Could this question (the wording, picture or its layout) be made clearer? If so, in what way?
- Was it easy or difficult to answer the question?
- What things were you thinking about when you answered the question?

- [Note additional questions/probes as relevant for each round of interviews]

More Spontaneous Probes:

- I noticed you were spending some time with that question – can you tell me what you were thinking about?
- You answered that very quickly, why is that?
- You answered ______ - in your own words, what does that mean?
- How sure are you of your answer?
- How did you feel about answering this question?
- I noticed you were looking here (and there), what were you thinking?

ASCOT
adult social care outcomes toolkit

University of Kent

ASCOT domain: safety outside of home

Let the participant read (out) the question and answers, and give an answer.

**Insert latest round version of the safety outside of home domain question and answers to be used in each round of interviews**

Questions/probes (if needed):

- How would you answer this question? / Which answer would you choose?
- Why? / What were you thinking about when you chose that answer? / Why did you choose this answer rather than …?
- Could this question (the wording, picture or its layout) be made clearer? If so, in what way?
- Was it easy or difficult to answer the question?
- What things you thinking about when you answered the question?

- [Note additional questions/probes as relevant for each round of interviews]

More Spontaneous Probes:

- I noticed you were spending some time with that question – can you tell me what you were thinking about?
- You answered that very quickly, why is that?
- You answered ______ - in your own words, what does that mean?
- How sure are you of your answer?
- How did you feel about answering this question?
- I noticed you were looking here (and there), what were you thinking?

ASCOT domain: social participation

Let the participant read (out) the question and answers, and give an answer.

**Insert latest round version of the social participation domain question and answers to be used in each round of interviews**

Questions/probes (if needed):

- How would you answer this question? / Which answer would you choose?
- Why? / What were you thinking about when you chose that answer? / Why did you choose this answer rather than …?
- What time period were you thinking of when you answered this question?
- Could this question (the wording, picture or its layout) be made clearer? If so, in what way?
- Was it easy or difficult to answer the question?
-
- What things were you thinking about when you answered the question?

- [Note additional questions/probes as relevant for each round of interviews]

More Spontaneous Probes:

- I noticed you were spending some time with that question – can you tell me what you were thinking about?
- You answered that very quickly, why is that?
- You answered ______ - in your own words, what does that mean?
- How sure are you of your answer?
- How did you feel about answering this question?
- I noticed you were looking here (and there), what were you thinking?

ASCOT domain: occupation (how you spend your time)

Let the participant read (out) the question and answers, and give an answer.

**Insert latest round version of the occupation domain question and answers to be used in each round of interviews**

Questions/probes (if needed):

- How would you answer this question? / Which answer would you choose?
- Why? / What were you thinking about when you chose that answer? / Why did you choose this answer rather than …?
- Could this question (the wording, picture or its layout) be made clearer? If so, in what way?
- Was it easy or difficult to answer the question?
- What things were you thinking about when you answered the question?

- [Note additional questions/probes as relevant for each round of interviews]

More Spontaneous Probes

- I noticed you were spending some time with that question – can you tell me what you were thinking about?
- You answered that very quickly, why is that?
- You answered ______ - in your own words, what does that mean?
- How sure are you of your answer?
- How did you feel about answering this question?
- I noticed you were looking here (and there), what were you thinking?

ASCOT domain: dignity

Let the participant read (out) the question and answers, and give an answer.

**Insert latest round version of the dignity domain question and answers to be used in each round of interviews**

Questions/probes (if needed):

- How would you answer this question? / Which answer would you choose?
- Why? / Who were you thinking about when you chose that answer? / Why did you choose this answer rather than …?
- How did you feel about answering this question?
- Could this question (the wording, picture or its layout) be made clearer? If so, in what way?
- Was it easy or difficult to answer the question?
- Could this question be asked differently / better?
- What do you think the question is trying to find out?
- What things were you thinking about when you answered the question?

- [Note additional questions/probes as relevant for each round of interviews]

More Spontaneous Probes:

- I noticed you were spending some time with that question – can you tell me what you were thinking about?
- You answered that very quickly, why is that?
- You answered ______ - in your own words, what does that mean?
- How sure are you of your answer?
- I noticed you were looking here (and there), what were you thinking?

End of the interview

Is there anything else you would like to mention before we finish the interview?

Thank you for taking time out of your day to speak with me.
