# Supplementary material for: Enhancing quality of life measurement: adapting the ASCOT easy read for older adults accessing social care
Source: Qual Life Res. 2024 Sep 26;34(1):189–200. doi: 10.1007/s11136-024-03791-0 (PMC11802674; doi:10.1007/s11136-024-03791-0)
Supplement: Supplementary file 4 — Supplementary file4 (PDF 104 KB) [file 11136_2024_3791_MOESM4_ESM.pdf]

**Coding examples using Tourangeau's four stage response model**

| **Response stage issue coded** | **Transcript** |
|---|---|
| Comprehension issue | Home cleanliness and comfort question: SR03<br><br>Q: Yes, I have.  So we'll have a look at this one then. [Accommodation question].<br><br>A: All right.  Well, it's – I don't get asked to do the washing up but I do it and – because I think it's a way of saying thank you, which is one thing.  And there's been no harassing of me and I think that's an important thing because they were colleagues and so I decided that instead doing the way I was just about starting to look at them, was to, um, oh, God, what was I talking about?  Er, sort of – what was I starting –<br><br>Q: You were talking about washing up.<br><br>A: Washing up, that's it.  I – it's – it was in one of the hotels.  Well, it was a church thing actually.  And I said to the person in charge, and I can't remember exactly what it was now, but, um, yeah, I had to – and I say, well, I'll make, um, I'll sort of clean the cutlery for you tomorrow.  And I've been doing it ever since.  So it [both laugh] – and I hate it [both laugh].  So it's, um –<br><br>… I don't – I feel people are just very warm and comfortable, kind of thing.  I don't feel that there are any of that's – that type of thing, sort of fairly easy-going.  But there is, you know, there is a point at which, you know, you got to be able to recognise, yeah, you know, what we're doing here, for example, you know, but, um, and I think that's a fair point I'd, you know, sort of thing [laughs]. |
| Recall issue | Food and drink question: SR02<br><br>A: I don't know.  What do I have for breakfast?  I can't think what I have for breakfast.  What do I have for breakfast?<br><br>Q: Do you have toast?  Or are you not sure?<br><br>A: No.  I don't know. |

| | |
|---|---|
| Judgement issue | Food and drink question: JC02<br><br>Q: Fine, okay, well, that's great. Let's have a look at the next one.<br><br>A: [Pause] This is a difficult one to some extent. Yes, I – I'm fed well but, I mean, when I occasionally ask for, I don't know what, coming back to you, you know, for example, I quite like the idea when someone recommended to me a particular meat pie shop.<br><br>Q: Okay.<br><br>A: But I would like meat pies and I mentioned it and said it but it just doesn't – that doesn't happen. I find that a bit annoying, that's about the only annoying thing I've got, yeah. But no, but in terms of having enough, yes. But from that point of view, in a way it's a little bit limited in that sandwiches are common. Okay, I understand that but, I mean, if there's one thing which annoys me, is that I can't ask for something, particularly like meat pies, they just never appear.<br><br>Q: So, bearing that in mind, where do you think you would – what box do you think you would tick for that answer?<br><br>A: [Pause] I suppose it would be the second one, yeah.<br><br>A: Yet some of the food and drink I like when I want – it's not entirely true but that's it. Some of, I would think. Yeah, I think number three.<br><br>Q: You'd put number three?<br><br>A: Yeah. I get some of the food and drink I like when I want. Perhaps that's being over critical.<br><br>Q: So option three says, "I get some of the food and drink I like when I want but not often enough. Sometimes I'm hungry or thirsty."<br><br>A: No, no, I wouldn't say there was that, ever, no, no. No, I'm being finicky, I suppose, to an extent.<br><br>A: No, that's going too far. |

| | |
|---|---|
| | Q: That's too much?<br><br>A: Absolutely, yeah.<br><br>A: …Um, well, I'd like something different, a bit more variety, you know, that – more variety would be something but not something I could really complain about.<br><br>A: [Pause] Hmm [pause]. I feel a bit guilty here. I get enough of the food and drink I like when I want, I can't honestly quite say that.<br><br>Q: Okay.<br><br>A: But I'm not sure – I'm not someone who constantly asks for it. But [pause] hmm. "Enough of the food and drink I like, when I want." Although I don't get – but because of the things – but I don't make a big song and dance about it. |
| Response Mapping issue | Occupation question: JC01<br><br>Q: … but you were saying, oh, I might cross it out and tick the second one because perhaps that's more your situation as it is now?<br><br>A: I'm able to... I'm able to do enough of the things I value. I guess, I suppose really, it's adaption but I'm able it says. It doesn't say I am able to spend my time as I want or can any of us do that?<br><br>Q: Yeah, well, that's the thing we were talking about –<br><br>A: Doing things I value or enjoy. So I suppose I should have ticked I am able to do enough of the things that I value or enjoy.<br><br>Q: Okay.<br><br>A: Yeah. Or I do some of the things that I value or enjoy but not enough. I could tick that. |

| | |
|---|---|
| | Q: So you could tick two or three maybe?<br><br>A: Yeah. Three, so far.<br><br>Q: Okay. So you wouldn't put yourself there, you wouldn't put yourself at the bottom line?<br><br>A: Not the bottom one. The first three then, yeah.<br><br>Q: Okay. Is it difficult to choose one of those between the first three?<br><br>A: Yeah. |
