# Supplementary material for: Enhancing quality of life measurement: adapting the ASCOT easy read for older adults accessing social care
Source: Qual Life Res. 2024 Sep 26;34(1):189–200. doi: 10.1007/s11136-024-03791-0 (PMC11802674; doi:10.1007/s11136-024-03791-0)
Supplement: Supplementary file 5 — Supplementary file5 (PDF 718 KB) [file 11136_2024_3791_MOESM5_ESM.pdf]

ASCOT Easy Read Occupation question for people with IDD and/autism - prior to consultation with Working Group

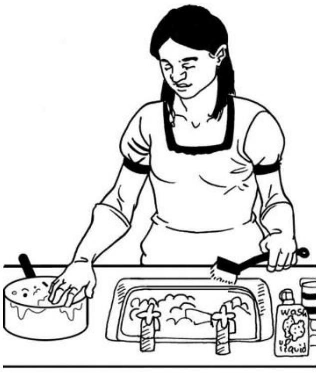

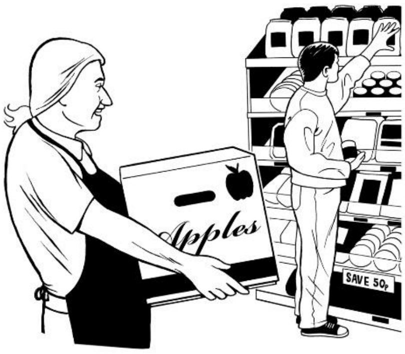

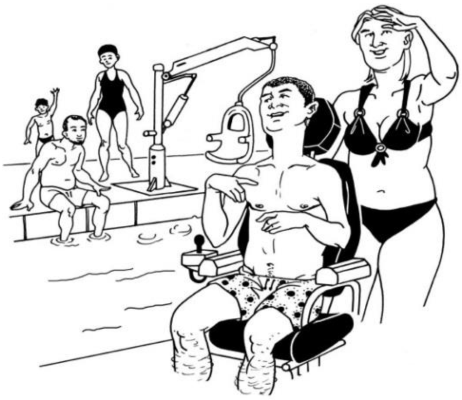

This question is about how you spend your time.

Think about all the things you do during the day. You could think about:

- Your free time.
- Going to work, college, or volunteering.
- Housework.

Think about if:

- You can choose the things you do.
- You enjoy the things you do.
- You have enough things to do.

How do you feel about the way you spend your time?

Please tick (✓) 1 box

I spend my time how I want. It is great. ☐ 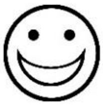

I do enough of the things I like. It is OK. ☐ 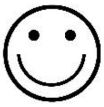

I do some of the things I like. But I would like to do more. ☐ 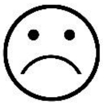

I do not do the things I like. It is really bad. ☐ 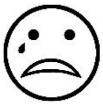

© PSSRU at the University of Kent

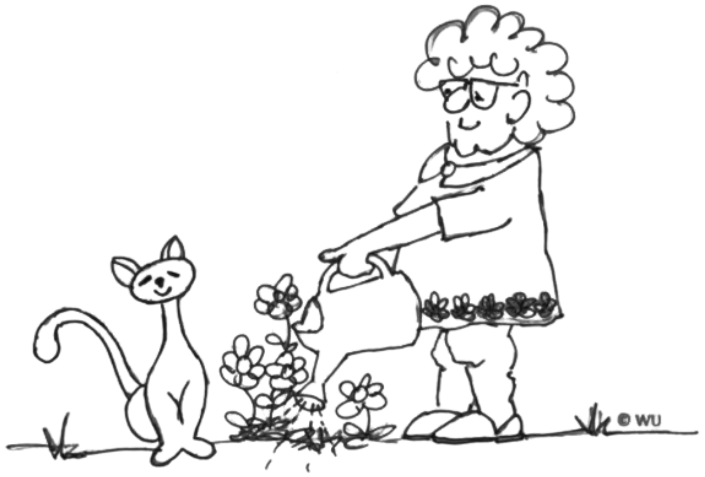

This question is about how you spend your time.

Think about all the things you do during the day. This could be your free time, volunteering or helping others, and doing housework.

Think about if:

- You can choose the things you do.
- You enjoy the things you do.
- You have enough things to do.

How do you feel about the way you spend your time?

Please tick (✓) 1 box

I'm able to spend my time as I want, doing things I value or enjoy. ☐

I'm able to do enough of the things I value or enjoy. ☐

I do some of the things I value or enjoy, but not enough. ☐

I don't do anything I value or enjoy. ☐

© PSSRU at the University of Kent
