# Supplementary material for: Enhancing quality of life measurement: adapting the ASCOT easy read for older adults accessing social care
Source: Qual Life Res. 2024 Sep 26;34(1):189–200. doi: 10.1007/s11136-024-03791-0 (PMC11802674; doi:10.1007/s11136-024-03791-0)
Supplement: Supplementary file 9 — Supplementary file9 (PDF 245 KB) [file 11136_2024_3791_MOESM9_ESM.pdf]

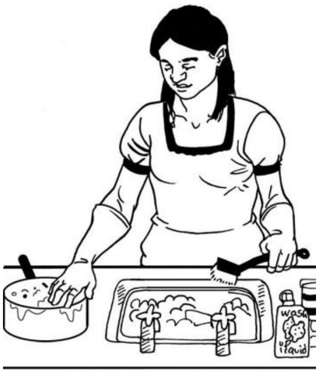

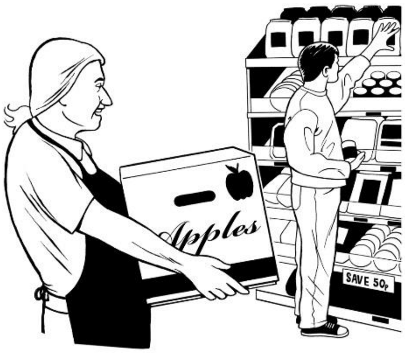

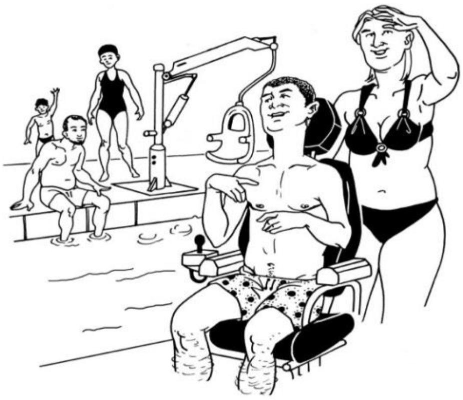

This question is about how you spend your time.

Think about all the things you do during the day. You could think about:

- Your free time.
- Going to work, college, or volunteering.
- Housework.

Think about if:

- You can choose the things you do.
- You enjoy the things you do.
- You have enough things to do.

How do you feel about the way you spend your time?

Please tick (✓) 1 box

I spend my time how I want. It is great. ☐ 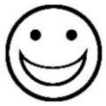

I do enough of the things I like. It is OK. ☐ 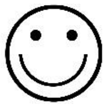

I do some of the things I like. But I would like to do more. ☐ 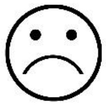

I do not do the things I like. It is really bad. ☐ 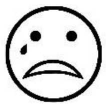

This question is about how you spend your time.

Think about all the things you do during the day.

This could be helping others, doing housework, or leisure activities like hobbies, watching TV and reading.

Think about if:

You can choose the things you do.

You enjoy the things you do.

You have enough things to do.

How do you feel about the way you spend your time?

Please tick only 1 box ☑

☐ I'm able to spend my time as I want, doing things I value or enjoy.

☐ I'm able to do enough of the things I value or enjoy.

☐ I do some of the things I value or enjoy, but not enough.

☐ I don't do anything I value or enjoy.
